# Psychometric validation of the fit body scale for assessing muscularity related body dissatisfaction in Iranian adults

**DOI:** 10.1038/s41598-026-46174-w

**Published:** 2026-07-01

**Authors:** Fatemeh Sadat Raeisian, Hamid Poursharifi, Sepideh Rajezi Esfahani, Milad Kadkhoda, Bahar Dehghanpour Hanzaei, Mohammadreza Davoudi

**Affiliations:** 1 Department of Clinical Psychology, School of Behavioral Sciences and Mental Health (Tehran Institute of Psychiatry), Iran University of Medical Sciences, Tehran, Iran; 2 Department of Clinical Psychology, Faculty of Behavioral Sciences, University of Social Welfare and Rehabilitation Sciences, Tehran, Iran; 3 Faculty of Medicine, Clinical Epidemiology, Memorial University of Newfoundland, St. John’s, Newfoundland Canada; 4 Department of Clinical Psychology, Faculty of Behavioral Science, University of Social Welfare and Rehabilitation Sciences, Tehran, Iran

**Keywords:** Body image, Muscularity ideal, Fit body scale, Psychometric properties, Body dissatisfaction, Psychology, Human behaviour

## Abstract

The Fit Body Scale is a newly developed scale that measures body dissatisfaction with muscularity ideals. It has gender-specific versions, the Female Fit Body Scale (FFITBS) and the Male Fit Body Scale (MFBS). The current study examined the psychometric properties of the Fit Body Scale in Iranian women and men. A total of 667 females and 226 males from the Iranian population completed a battery of tools, including FFITBS, MFBS, Drive for Muscularity Scale (DMS), Eating Attitude Test (EAT-26), Body Appreciation Scale (BAS), and Yale-Brown modified Obsessive-Compulsive Scale for Body Dysmorphic Disorder (YBOCS-BDD). We examined the 2-week test-retest reliability, and the results showed a satisfactory Intraclass Correlation Coefficient (ICC) for both FFITBS and MFBS. For both genders, relationships between body mass index (BMI) and current body shape selected by participants from nine body shapes in the FFITBS and MFBS were positive, demonstrating the scales’ concurrent validity. In addition, the correlations of DMS with FFITBS and MFBS were positive, indicating convergent validity. The correlation between EAT-26 and FFITBS was significant, but its correlation with MFBS was insignificant. The significant negative correlation between BAS and FFITBS indicates divergent validity of FFITBS, but no correlations were found with MFBS. The study results suggest that the FFITBS and MFBS are psychometrically valid tools for measuring body dissatisfaction regarding the muscularity ideal among the Iranian population.

## Introduction

Body image refers to the mental representation we have of our body’s size, shape, and form, as well as our emotional responses to these characteristics and our individual body parts^1^. It is a multidimensional construct with perceptual, cognitive, and emotional dimensions. The perceptual dimension of body image includes how a person sees and describes the appearance and function of their body (estimate of body size or weight); the cognitive dimension evaluates the thoughts related to the appearance and function of the body; the emotional dimension emphasizes the feelings and emotions of the person regarding the appearance and function of their body; the behavioral dimension relates to the actions stemming from an individual’s perception, thoughts, and feelings of their body’s appearance and functionality^2,3^.

Cultural norms significantly influence individuals’ perceptions of their bodies through beauty standards and ideal bodies. These norms are historically, socially, and religiously constructed, shift over time, and differ markedly across societies^4^. In Western culture, thinness is often regarded as the ideal body type for women, while muscularity is considered the ideal for men. These ideals are promoted through media, fashion, and advertising, creating a pervasive culture in which deviation from the perfect leads to body dissatisfaction and feelings of inadequacy^5^. In contrast, in past decades, many Eastern cultures—including Iran—traditionally favored women with higher adiposity or greater body mass, as these traits were associated with health and fertility. Consequently, women’s body satisfaction tended to be higher in these cultural contexts^6^. Additionally, a muscular body was considered ideal for men, aligning with traditional masculine roles emphasizing strength^7^. However, in recent years, globalization, the expansion of the Internet, and social networks have introduced Western cultural values ​​regarding the ideal body to Eastern countries, including Iran. As a result, the ideal body for women has shifted toward thinness, while the ideal for men has remained on muscularity^8^. Following this change, dissatisfaction with the body has increased in non-Western countries like Iran, as research shows that most men and women are dissatisfied with their bodies^9^.

In the past twenty years, most research on body image and body dissatisfaction has primarily focused on the thin-ideal among women. Thin-ideal (the desire to have a thin body rather than being fat) is considered a key factor in body dissatisfaction and is linked to health-threatening behaviors such as restrictive eating, intentional vomiting, and excessive exercise^10^. While most assessments of body dissatisfaction in women emphasize the pursuit of an idealistically thin body, recent evidence suggests that sociocultural ideals of women’s physical attractiveness have evolved from merely valuing thinness to embracing a thin-muscular ideal^11,12^. The thin-muscular ideal combines thinness with a compact level of muscularity. Various terms used in research, such as “athletic”, “fit”, or “toned”, refer to this ideal. Despite the different labels, they all share a common characteristic: they emphasize moderate muscularity with low body fat (i.e., leanness)^13^. The musculature considered attractive in women differs from that in men. Men are expected to have larger, more defined muscles (in addition to leanness)^7^. Conversely, women tend to prefer a musculature that is neither excessively large nor bulky, but rather somewhat toned and elongated. This is because this physique is consistent with the traditional and desirable female shape(slim)^14^. Evidence shows that women prefer a body type that is both thin and visibly muscular rather than one that is extremely thin or highly muscular alone, reflecting a preference for the thin-muscular ideal^15^.

Research on body dissatisfaction and its antecedents and consequences has mainly focused on women, particularly their desire to control their weight and body shape. This emphasis perpetuates the misconception that body image issues are exclusive to women. However, evidence shows that men also struggle with negative thoughts and feelings about their bodies^16^. A body characterized by a low body fat percentage and large, defined muscles is considered ideal for men^17^. Sociocultural factors contribute to men’s inclination towards muscularity, which is regarded as a key aspect of masculinity. The pressure to be muscular has increased to reinforce stereotypical masculine characteristics, such as strength and power, especially at a time when traditional male roles, such as breadwinner, are less exclusive and have lost their value as a gender differentiator^18^.

The tripartite influence model explains that key sociocultural factors, such as social media, family, and peers, promote body image ideals (such as the muscularity ideal) and pressure individuals to conform to these standards. This process will be facilitated via two main mechanisms: internalization and social comparison. Internalization involves individuals accepting and valuing messages about appearance. Meanwhile, social comparison *involves* comparing one’s body to sociocultural ideal body standards. Given that these ideals are unattainable for most individuals, internalization and social comparison highlight the differences between the individual’s body and the perfect body, ultimately leading to body dissatisfaction^19,20^.

Body dissatisfaction is widespread, affecting approximately 11–72% of women and 8–61% of men worldwide‌‌^21^. In Iran, its prevalence has increased notably, with 60% of women and 30% of men reporting dissatisfaction with their bodies ^22^. This dissatisfaction has significant negative consequences for both physical and mental health. Regarding physical health, some individuals may engage in risky behaviors such as anabolic steroid overuse to gain muscle, extreme dieting, and excessive exercise to lose fat ^23^. Regarding mental health, chronic body dissatisfaction can lead to persistent low mood, decreased self-esteem ^24^, increased risk of depression, impaired daily functioning, and reduced quality of life^25^. Additionally, it is associated with increased risk of eating disorders^26^ and leads to appearance-related avoidance behaviors, resulting in social isolation, loneliness, and negative mental health outcomes^27,28^. Therefore, assessing and diagnosing body dissatisfaction is crucial to enable timely preventive and treatment interventions^29^.

Body dissatisfaction is commonly assessed through questionnaires that evaluate the person’s level of acceptance regarding their weight and body shape, and graphical tools containing images of human body shape, such as figure rating scales^30^. One of the most recognized tools is Stunkard’s Figure Rating Scale (SFRS)^31^, which is a valid scale for measuring body dissatisfaction related to perceptions of thinness and obesity in both men and women^32^. This scale includes schematic figures representing a range of body types, from very thin to fat. The difference between an individual’s ideal body shape (IBS) and current body shape (CBS) indicates their level of body dissatisfaction (IBS-CBS)^33^. The SFRS has been widely used in non-Western countries, such as Saudi Arabia, China, Kuwait, and Iran, to effectively assess body dissatisfaction. Research suggests that this scale has acceptable validity and reliability in these cultural contexts, allowing researchers and health professionals to use this scale to gain a better understanding of body dissatisfaction in non-Western societies^34–37^. However, certain limitations regarding SFRS exist; one significant drawback is that it only measures the drive to be thin while ignoring the drive for muscularity, which can be an essential factor in body dissatisfaction for both men and women^38–40^.

Some tools measure drive for muscularity, but they also have limitations. One of the most common tools is the Somatomorphic Matrix(SM), which measures body dissatisfaction related to thinness and muscularity. The SM is a computer program designed to assess individuals’ body image using photos and accurate real-life measurements, primarily of Caucasians. Each image in the program represents a specific body type, with its fat percentage and muscle mass determined by scientific calculations. A key feature of the Somatomorphic Matrix is that it allows individuals to select their “current” body (the image they think reflects their current body appearance) and their “ideal” body (the body they aspire to have). The program then compares these images, helping individuals better understand the difference between their perceived and actual body^41^. This tool has been used in various studies, including investigations of differences in body image across Western and Eastern cultures^42^. However, it has a significant limitation: it does not have acceptable reliability^43,44^. The Drive for Muscularity Scale (DMS) is another instrument that measures attitudes and behaviors related to the desire for a muscular body. This self-report measure consists of 15 items; each scored on a 6-point Likert scale (from never to always). The highest score indicates greater tendency towards muscularity^45^. This instrument has been widely used in various contexts, including Iran, and has shown good validity and reliability^32,46^. However, this assessment relies heavily on verbal skills and may not be appropriate for individuals with low literacy levels^38,47^.

Visual scales have advantages over language-based questionnaires, helping to address some of their limitations. First, unlike language-based questionnaires, individuals can use visual scales regardless of their literacy levels. This makes them suitable for diverse populations, including those who are illiterate or have cognitive impairments that make processing verbal statements challenging. Second, the visual format of these assessments aligns more closely with participants’ perceptions of their physical appearance^48^. Third, while language-based questionnaires typically assess cognitive and behavioral dimensions of body image, visual scales specifically focus on the perceptual dimension of this construct. Additionally, visual scales require less time to administer than language-based questionnaires^49^. To leverage these advantages and address the shortcomings of conventional instruments, Ralph-Nearman & Filick developed the Female Fit Body Scale (FFITBS) and the Male Fit Body Scale (MFBS). These scales depict a nine-item set of shapes of female (in FFITBS) and male (in MFBS) bodies, with all forms varying from lean to very muscular, allowing for an assessment of body dissatisfaction regarding muscular ideals^38,47^.

### Current study

Cultural values and the environment in which a person lives play a critical role in shaping body image and the development of body dissatisfaction. The degree and type of body dissatisfaction experienced can differ significantly among various cultural groups^50,51^. For instance, there are substantial differences between Eastern countries like Iran and Western countries. In Iran, Islamic regulations like hijab require both men and women, particularly women, to cover their bodies. This reduced exposure may influence how Iranian men and women conform to body ideals^52,53^. Nevertheless, in recent years, body dissatisfaction regarding the muscularity ideal and its negative consequences has increased in Iran^22,46^.This growing trend highlights the need for early identification and design of effective interventions. However, existing instruments (such as the SFRS and DMS) used in domestic research to assess body dissatisfaction have significant limitations, as discussed previously, and no instrument currently exists that can rapidly and separately assess body dissatisfaction regarding muscular ideals in men and women. Therefore, evaluating the psychometric properties of new measures, specifically Female and Male Fit Body Scales (FFITBS and MFBS), in this cultural context is essential. The present study examines the psychometric properties of the Female and Male Fit Body Scales (FFITBS and MFBS) in an Iranian sample. The following hypotheses are formulated:

- **Test–retest reliability**: Building on the original studies demonstrating acceptable test-retest reliability of the FFITBS and MFBS in assessing current body shape, ideal body shape, and body dissatisfaction scores^38,47^, the present study hypothesized that FFITBS and MFBS scores on these three constructs would be positively and significantly correlated between the initial test and a two-week retest.
- **Concurrent validity**: Based on the findings of previous studies showing that individuals with lower body mass index (BMI) tend to choose leaner shapes as their current body shape^38,47^, this study hypothesized a significant positive correlation between participants’ body mass index (BMI) and their self-selected current body shape on both scales.
- **Convergent validity with eating attitudes test-26 (EAT-26)**: Given the studies indicating that individuals with higher body dissatisfaction are more likely to develop eating disorders^54,55^, the present study hypothesized that body dissatisfaction scores from the FFITBS and MFBS would be positively and significantly correlated with scores on the Eating Attitudes Test-26 (EAT-26).
- **Convergent validity with drive for muscularity scale (DMS)**: Consistent with previous studies, evidence that individuals with higher body dissatisfaction tend to prefer a more muscular body^56,57^, this study hypothesized that body dissatisfaction scores from FFITBS and MFBS would be positively and significantly correlated with scores on the Drive for Muscularity Scale (DMS).
- **Divergent validity**: Based on the studies that found an association between higher levels of body appreciation and lower levels of body dissatisfaction^58,59^, we hypothesized a significant negative correlation between FFITBS and MFBS body dissatisfaction scores and scores on the Body Appreciation Scale (BAS).

## Methods

### Participants

The current research was a cross-sectional study conducted in Iran from July 27th, 2023, to December 17th, 2023. Eight hundred ninety-three participants, including 667 (74%) women and 226 (26%) men, were selected using convenience sampling from the general population. The ages of the participants ranged from 18 to 30 years (M = 23.73, SD = 3.88 for women and M = 23.96, SD = 3.70 for men). The BMI ranged from 14.28 to 36.98 (M = 22.34, SD = 3.62) for women and 16.62 to 43.83 (M = 24.05, SD = 3.89) for men. The demographic information of the participants is presented in Table 1. According to this table, the majority of both men and women participants were unemployed, single, Persian, and had a bachelor’s degree.

For the assessment of test–retest reliability (as detailed in the Procedure section), a subsample of 60 participants —29 women (M _age_ = 25.38 years, SD = 3.75; M _BMI_ = 22.90, SD = 3.51) and 31 men (M _age_ = 25.29 years, SD = 2.84; M _BMI_ = 24.57, SD = 2.96) — were invited to complete the scales on two occasions separated by a two-week interval.

**Table 1:** Frequency distribution of participants’ demographic characteristics.

Variable		Females [*n* (%)]	Males [*n* (%)]	χ2
Occupational Status	Employed	265(39.7)	131(58)	*p* < 0.05
	Unemployed	402(60.3)	95(42)	
Marital Status	Married	145(21.8)	14(6.2)	*p* < 0.05
	Single	503(75.4)	207(91.6)	
	Divorced	19(2.8)	5(2.2)	
Ethnicity	Persian	377(56.5)	109(48.2)	*p* > 0.05
	Turk	121(18.7)	50(22.1)	
	Lor	67(10)	22(9.7)	
	Kurd	89(13.3)	43(19)	
	Arab	13(1.9)	2(0.9)	
Educational Status	Below Diploma	11(1.6)	3(1.3)	*p* > 0.05
	Diploma	219(32.8)	76(33.6)	
	Bachelor	307(46)	103(45.6)	
	Master	109(16.3)	39(17.3)	
	Doctorate and above	21(3.1)	5(2.2)	

Participants were eligible for inclusion if they provided informed consent, were aged 18–30 years, and resided in Iran. Exclusion criteria included a history of body dysmorphic disorder (BDD), assessed using the Yale-Brown Obsessive-Compulsive Scale modified for BDD (YBOCS-BDD), as individuals with BDD tend to exhibit excessive preoccupation with appearance^60^, potentially acting as outliers in body image research. In addition, pregnant women were excluded due to the significant physical changes experienced during pregnancy^61^, which can temporarily increase body dissatisfaction and distort the findings.

### Cross-cultural translation of the fit body scale

The Fit Body Scale was developed to measure body dissatisfaction and includes distinct gender-specific versions for males and females. This scale comprises nine body images, from lean to very muscular, along with two questions. The linguistic validation of the Fit Body scale from English to Persian followed guidelines for cross-cultural adaptation and validation of psychological instruments^62^. The process began with two Persian speakers who were fluent in English translating the two scale questions from English to Persian, resulting in two translated versions, referred to as I and II (forward translation). One of the translators was a doctoral candidate specializing in body image, and the other was an English teacher with 10 years of experience who did not have a background in psychology. The article’s first author met with the translators to combine Forms I and II, resulting in the development of Form III (translation synthesis). Form III was back-translated into English by two other English translators who did not specialize in behavioral sciences, creating Form IV (backward translation). The researchers of the present study compared Form IV and the original version of the fit body scale to identify discrepancies in the translation process, and by making minor changes by the people mentioned above, it was confirmed that the two versions are the same. As a result, the final form was created.

### Pilot study

A pilot study was conducted with 30 university students (15 women and 15 men) aged 18–30 years to evaluate the comprehensibility and clarity of the translated scales. Participants were provided with the final version of the scales and asked to read each item aloud, paraphrase its meaning in their own words, identify any confusing or ambiguous terms, and suggest alternative wording where necessary. This cognitive interviewing approach allowed us to assess how items were interpreted and ensure they were free of ambiguity. During this process, respondents indicated that the phrase “ideal body shape” was too vague to be interpreted clearly. Consequently, “ideal body shape” was specified as “ideal body shape (in terms of appearance)”. A brief follow-up test with five additional participants confirmed that the revised items were now understood as intended.

### Study procedure

This study was conducted through an online survey website called Porsline. All questionnaires were presented on the website, and their links were shared via social media platforms, including Telegram, WhatsApp, Instagram, and LinkedIn. After participants agreed to participate in the study and responded to demographic questions by gender, they were directed to either the FFITBS or the MFBS. The participants then answered the remaining questionnaires. To minimize the influence of biased responses to first and later questions on participants’ answers and to ensure accurate results, the questionnaires were presented in a randomized order using the platform’s randomization option.

A total of 1047 people volunteered to answer the questionnaires. The participants were screened for exclusion criteria: 18 women were excluded because they were pregnant, and 136 participants (108 women and 28 men) were excluded for body dysmorphic disorder (YBOCS-BDD scores > 20). Finally, 893 participants remained, and their data were analyzed.

In the test-retest reliability study, 60 participants (described in the participant section) were randomly selected from the original sample (*N* = 893) using the Randomizer.org website. These participants received an email invitation that included a brief explanation of the retest purpose and procedure, and they were asked to confirm their participation. All 60 invited participants provided consent. Two weeks after the initial assessment, they received an email with a link to the second-stage questionnaire and a unique four-digit identification code (author-generated). Code entry was mandatory, and all participants entered the correct code, resulting in 100% response matching.

### Ethical approval and consent from participants

Every procedure used in this study adhered to the ethical standards outlined in the Declaration of Helsinki of 1964 and subsequent amendments. All participants provided informed consent in accordance with these standards. This study was approved by the Ethics Committee of the University of Social Welfare and Rehabilitation Sciences (IR.USWR.REC.1401.031).

### Measures

#### Demographic information

Participants self-reported their age, gender, occupational status, marital status, educational status, ethnicity, height, and weight (used to calculate their BMI). Female participants also reported their pregnancy status.

#### Fit body scale

The Fit Body Scale was developed by Ralph-Nearman & Filik and has two versions for females and males, called the Female Fit Body Scale (FFITBS) and the Male Fit Body Scale (MFBS) (see the main study for the scales)^38,47^. This scale consists of nine body shapes representing varying levels of muscularity, with the first representing a lean body and the ninth representing a very muscular physique. To measure body dissatisfaction regarding muscularity ideal using these scales, the participants were asked to choose two shapes: 1- The shape that represents their current body (a person’s perception of their body) (CBS). 2- The shape representing their ideal body (the body they would like to have) (IBS). The difference between ideal and current body shape (IBS-CBS) indicates body dissatisfaction. Positive and negative signs show the direction of body dissatisfaction. If the scores were summed to 0, it would mean that participants have no dissatisfaction; a positive score indicates body dissatisfaction and an urge to have a more muscular body, and a negative score suggests body dissatisfaction and a preference for a leaner body. In the original development and validation studies of the FFITBS and MFBS, both scales exhibited acceptable test-retest reliability and validity^38,47^.

#### Drive for muscularity scale (DMS)

The DMS is a 15-item questionnaire developed by McCreary and Sasse that examines attitudes and behaviors related to how preoccupied people are with increasing their muscularity. Each item is scored on a 6-point scale from 1 (never) to 6 (always)^45^. The DMS has two subscales: Muscularity Attitude (7 items, score 7–42) and Muscularity Behavior (8 items, score 8–48). A person’s overall score on this scale ranges from 15 to 90, with higher scores indicating a greater drive for muscularity. In the current study, only the total DMS score was used for analyses. In the Persian version of the DMS, one question was removed because it did not have a significant factor loading on either of the two scale factors. Therefore, the final version includes 14 questions, with a total score ranging from 14 to 84. This scale has good internal consistency and acceptable validity in the Iranian population^46^. The present study assessed the DMS’s internal consistency. Cronbach’s alpha coefficient was 0.83 for the entire sample, 0.81 for women, and 0.84 for men.

#### Eating attitudes test (EAT-26)

This self-reporting scale has 26 questions, each answered on a 6-point Likert scale from 1 (never) to 6 (always)^63^. Item responses are recoded to obtain the total score: scores 1–3 as 0, 4 as 1, 5 as 2, 6 as 3, except for item 26, which is reverse-scored (1 = 3, 2 = 2, 3 = 1, 4–6 = 0). The questionnaire consists of three subscales: Dieting (13 items, score 0–39), Bulimia and Food Preoccupation (6 items, score 0–18), and Oral Control (7 items, score 0–21). The total score is the sum of these three subscales and ranges from 0 to 78. A total score of 20 or higher indicates risk for an eating disorder^63^. In the current study, only the total score of the EAT-26 was used for analyses. The Persian version of the EAT-26 has acceptable internal consistency, test-retest reliability, and validity in the Iranian population^64^. In this study, Cronbach’s alpha coefficients were 0.75 for the total sample, 0.74 for women, and 0.77 for men, reflecting acceptable reliability.

#### Body appreciation scale (BAS)

The Body Appreciation Scale (BAS) was developed by Avalos et al. to measure the construct of body appreciation^65^. This single-factor scale consists of 13 items scored on a 5-point Likert scale (1 = never to 5 = always). The total score of this scale ranges from 13 to 65, with higher scores indicating greater body appreciation. The Persian version of the BAS demonstrated a single-factor structure with acceptable internal consistency and validity in the Iranian population^66^. In the present study, the internal consistency of the BAS was evaluated. Cronbach’s alpha coefficient was 0.94 for the entire sample, 0.94 for women, and 0.93 for men.

#### Yale-Brown modified obsessive-compulsive scale for body dysmorphic disorder (YBOCS-BDD)

The YBOCS-BDD is a 12-item self-assessment tool that measures the severity of symptoms of body dysmorphic disorder. On a Likert scale ranging from 0 (no symptomatology) to 4 (severe symptomatology), participants rate their agreement with each item. A participant’s total score on the YBOCS-BDD ranges from 0 to 48^67^. A score above 20 has been determined to be indicative of body dysmorphic disorder^68^. The Persian version of the YBOCS-BDD has acceptable internal consistency, test-retest reliability, and validity in the Iranian population^69^. In the present sample, the scale showed acceptable internal consistency, with Cronbach’s alpha values of 0.71 for the full sample, 0.71 for women, and 0.70 for men.

### Analysis

SPSS statistical software (version 23) was used for statistical analysis in this study. First, data screening was undertaken to investigate normality, missing values, and outliers. The normality of the data distribution was assessed using skewness and kurtosis. According to established scientific criteria, the data distribution is considered normal if the skewness and kurtosis values for the variables are within the range of -2 to + 2^70^. The distribution was considered normal since the calculated values for the data examined in this study were within this range. The mean and standard deviation were used to describe quantitative data, and frequencies, percentages, and chi-square tests were used to describe qualitative data.

Intraclass correlation coefficients (ICCs) were calculated to assess test–retest reliability, with values below 0.50 indicating poor reliability, 0.50–0.75 moderate reliability, 0.75–0.90 good reliability, and above 0.90 excellent reliability^71^. Concurrent validity was examined by calculating Pearson’s correlation between participants’ self-reported current body shape, selected on the FFITBS and MFBS, and BMI. Convergent validity was assessed by correlating dissatisfaction regarding muscularity ideal scores (derived from FFITBS and MFBS) with DMS and EAT-26 scores. In contrast, divergent validity was examined by correlating the identical scores with BAS scores.

## Results

### Descriptive analysis

Descriptive findings related to the Fit Body Scale are presented by gender in Table 2. According to this table, the mean body dissatisfaction score for males is positive, indicating that *they* tend to prefer more muscular bodies. In contrast, the sign of mean body dissatisfaction among females is negative, suggesting that they prefer leaner bodies.

**Table 2:** Descriptive statistics of current body shape, ideal body shape, and body dissatisfaction.

Variable	Females				Males			
	Actual range	Possible range	Mean	SD	Actual range	Possible range	Mean	SD
Current body shape	1 to 7	1 to 9	2.98	1.27	1 to 9	1 to 9	4.02	1.43
Ideal body shape	1 to 9	1 to 9	2.80	1.09	2 to 9	1 to 9	5.46	1.62
Body dissatisfaction	-4 to 5	-8 to 8	− 0.17	1.19	-2 to 6	-8 to 8	1.44	1.64

The frequency distribution of participants’ Body dissatisfaction is presented in Table 3. According to this table, most females tended to prefer a leaner body, while most males tended to prefer a more muscular body.

**Table 3:** The frequency distribution of participants’ Body dissatisfaction.

	Females [*n* (%)]			Males [*n* (%)]		
	0	+	-	0	+	-
Body dissatisfaction	241(36.1)	170(25.5)	256(38.4)	50(22.1)	155(68.6)	21(9.3)

0 = the absence of body dissatisfaction + = tendency towards having a more muscular body -= tendency to have a leaner body.

Descriptive findings related to the DMS, EAT-26, and BAS are presented by gender in Table 4.

**Table 4:** Descriptive statistics of DMS, EAT-26, BAS.

Variable		Females			Males	
	Range	Mean	SD	Range	Mean	SD
DMS	14 to 66	29.68	10.29	14 to 73	37.26	11.92
EAT-26	0 to 33	8.75	6.93	0 to 35	8.69	7.63
BAS	15 to 65	54.61	9.02	20 to 65	52.56	10.00

### Reliability of the fit body scale

Table 5 shows the ICC value for current body shape, ideal body shape, and body dissatisfaction. According to this table, the ICC values for current body shape, ideal body shape, and body dissatisfaction in both genders are greater than 0.7, indicating acceptable reliability.

**Table 5:** Test-retest reliability of the Fit Body Scale.

Variable	Females		Males	
	ICC	95% CI (ICC)	ICC	95% CI (ICC)
Current body shape	0.90	0.79-0.95	0.81	0.61-0.91
Ideal body shape	0.88	0.74-0.94	0.74	0.46-0.87
Body dissatisfaction	0.88	0.74-0.94	0.74	0.47-0.87

### Concurrent validity of the fit body scale

To assess concurrent validity, the correlation between participants’ perceived current body size, as indicated by their selection of one of the nine shapes on the FFITBS and MFBS, and their actual BMI was investigated. The results showed a positive and significant correlation between a participant’s actual BMI and their perceived current body size for females (*r* = 0.70, *p* < 0.05) and males (*r* = 0.57, *p* < 0.05) on the FFITBS and MFBS.

### Convergent validity of the fit body scale

The correlations of FFITBS and MFBS with both EAT-26 and DMS were examined to assess convergent validity. The Pearson correlation results are shown in Table 6. According to this table, there was a significant but weak correlation between FFITBS and EAT-26 in females. In males, there was no significant correlation between MFBS and EAT-26. FFITBS and MFBS demonstrated a positive, significant, but weak correlation with DMS in both females and males.

**Table 6:** Correlation of body dissatisfaction with EAT-26 and DMS.

Variable	Females		Males	
	*r*		*r*	
EAT-26	0.17^*^		0.04	
DMS	0.27^*^		0.29^*^	

^* *P* < 0.05^.

### Divergent validity of the fit body scale

The correlations among FFITBS, MFBS, and BAS were calculated to evaluate divergent validity. A significant negative correlation was found between FFITBS and BAS in females (*r* = -0.33, *p* < 0.05), which supports the divergent validity of the FFITBS. In contrast, no significant correlation was observed between MFBS and BAS in males (*r* = -0.12, *p* > 0.05).

## Discussion

The present research aimed to examine the psychometric properties of FFITBS and MFBS in the Iranian population. The findings suggest that the FFITBS and MFBS have acceptable reliability and concurrent, convergent, and divergent validity among this group.

These two scales measure body dissatisfaction regarding the muscularity ideal. This construct is conceptualized as a spectrum, with one end indicating a desire for a more muscular body and the other reflecting a preference for a leaner body. This study showed that in Iran, most women tend to prefer leaner bodies, and most men tend to prefer more muscular bodies, which aligns with the previous research results^38,47,72–74^.

As predicted in the first hypothesis, a significant correlation was found between the current body shape, ideal body shape, and body dissatisfaction scores at the initial assessment and the retest conducted two weeks later. The acceptable test-retest reliability coefficient indicates that FFITBS and MFBS are repeatable and that their test results are stable over time. This finding is consistent with the results reported in the original validation studies of the FFITBS and MFBS^38,47^.

In line with the second hypothesis, the study’s findings indicated a positive and significant relationship between BMI and current body shape in both men and women. This suggests that participants with a lower BMI tend to choose a leaner shape as their current body shape, a result that aligns with the findings reported in the original validation studies of the FFITBS and MFBS^38,47^.

The results of the present study showed a positive and significant relationship between FFITBS and EAT-26 in women, which confirms the third hypothesis and is consistent with previous studies that have shown a direct relationship between body dissatisfaction and eating disorder^54,55^. To explain this finding, it is essential to note that the ideal female body image today often combines both thinness and visible muscular tone. Crucially, this muscular ideal has not replaced the traditional emphasis on thinness — it has been added to it. In other words, women are now expected to be not only slim but also fit and toned, which can heighten body dissatisfaction^13^. This persistent societal pressure to achieve a thin ideal may drive some women to adopt harmful eating behaviors, such as food restriction, crash dieting, binge eating, or purging^15^.

The association between MFBS and EAT-26 in men was not statistically significant. Although this finding does not support the third hypothesis, it is consistent with some previous studies^47,75–77^. This lack of correlation may be due to the fact that the EAT-26 specifically measures concerns about fat and weight gain, as well as avoidance of eating, which are often associated with a desire to be thinner. In contrast, men tend to increase their muscle mass and have large muscles. Additionally, other behaviors not examined in this study might be more related to body dissatisfaction regarding the muscularity ideal, such as using performance-enhancing supplements^56^.

Consistent with the fourth hypothesis, the study’s results showed that FFITBS and MFBS were positively and significantly associated with DMS in both women and men. This finding is consistent with previous research that has found a positive relationship between drive for muscularity and body dissatisfaction^38,47,56,57^. Consistent with the fourth hypothesis, the study’s results showed that FFITBS and MFBS were positively and significantly associated with DMS in both women and men. This finding is consistent with previous research that has found a positive relationship between drive for muscularity and body dissatisfaction^38,47,56,57^. In line with the fourth hypothesis, the study’s results showed that FFITBS and MFBS were positively and significantly related to DMS in both women and men. This finding is consistent with previous research that has found a positive relationship between drive for muscularity and body dissatisfaction^38,47,56,57^. This correlation suggests that individuals with body dissatisfaction regarding the muscularity ideal (as measured by FFITBS and MFBS) are motivated to take action to increase their body musculature and desire greater muscularity (as measured by DMS)^39^.

The present study found a significant negative relationship between FFITBS and BAS in women, confirming the fifth hypothesis. This finding is consistent with previous studies showing that body appreciation is inversely related to body dissatisfaction^58,59^. To explain this finding, culture has a determining role in the perceived ideal body. Social factors, such as family, peers, and the media, convey culture’s messages about ideal weight, shape, and appearance, and pressure from these social factors leads to the internalization of these ideals. Since many people find it difficult or impossible to achieve the standards promoted by society through healthy means, this pressure frequently leads to feelings of body dissatisfaction^78^. In contrast, body appreciation can be one of the psychological factors that protect against sociocultural pressures in a way that the person internalizes fewer social norms and experiences less body dissatisfaction^2,79^.

Contrary to the fourth hypothesis, the relationship between MFBS and BAS was not significant among men. Several factors may explain this finding: first, the sample size of men in this study was small, which affected the correlation’s significance. Secondly, men generally exhibit higher body esteem than women. Men and women experience different levels of body appreciation influenced by social and cultural factors^80^. Sexual objectification plays a significant role in these differences; women are often viewed as objects, with their physical appearance determining their value. This phenomenon is reinforced by the media, advertising, and popular culture, leading to self-objectification. Self-objectification occurs when women internalize an objectified view of themselves and come to see themselves as objects. As a result, women may constantly monitor their bodies, compare them to unrealistic ideals, and feel ashamed of their appearance^81,82^. Studies show that women in Eastern cultures, such as Iran, especially of those of university age, are more exposed to sexual objectification than men, which can lead to a lower appreciation of their bodies, particularly if they do not conform to ideals of attractiveness^83–85^. In contrast, ideal body norms for men are more flexible, and men are under less societal pressure to achieve them than women. They place more value on non-physical attributes such as their personality or success, which can lead to higher body appreciation^81^.

The present study has several limitations. First, body mass index was calculated from participants’ self-reported height and weight. Although self-reported height and weight are widely used in body image research, it is considered a limitation because some individuals may misreport their height and weight and over- or under-report their actual height and weight^86^. Therefore, it is suggested that the researcher measure height and weight in future studies to reduce the possibility of error.

Second, the study was conducted online, which meant that participants who completed the surveys often shared specific characteristics or motivations. These may include a willingness to cooperate, a tendency to express opinions, an interest in the research topic, or greater comfort with using online platforms. As a result, these participants may differ from those who chose not to participate. Additionally, younger and more educated people are usually more likely to participate in these surveys due to their greater familiarity with technology and online platforms^87^. This can affect the generalizability of the findings, as people from different age groups and educational levels who are less familiar with online surveys may be overlooked. Future studies should therefore employ quota sampling based on age, education level, and digital literacy, and collaborate with local centers (e.g., adult education programs and health clinics) to distribute paper-and-pencil questionnaires and conduct in-person interviews. These strategies will ensure adequate representation of individuals with lower educational attainment and those with limited digital access or proficiency.

Third, this study collected data from the general population, so the results may not be generalizable to the clinical population, such as individuals with eating disorders and body dysmorphic disorder. It is particularly important to consider athletes, such as male and female bodybuilders, who may prioritize muscle gain, as well as models who tend to be more concerned with their appearance. Therefore, future research should examine the psychometric properties of the FFITBS and MFBS within these specific populations.

Fourth, in this study, employment frequency and marital status differed between men and women. This disparity may reflect genuine differences between Iranian men and women regarding employment and marital status. However, this difference may also result from the sampling method used or the fact that the study was conducted online. Since these factors can affect the results, it is recommended that future studies use random sampling methods, such as cluster or stratified sampling, to ensure that the sample more accurately reflects the community’s characteristics.

Fifth, data collection relied on self-reporting. Therefore, honesty, accuracy, and recall bias may have influenced the information collected. Future research should adopt a multimethod approach that integrates ecological momentary assessment via smartphone prompts with clinician-administered interviews and objective measures, such as 3D body scanning, to enhance the reliability of self-report data and minimize bias.

Sixth, while the BDD-YBOCS is validated for measuring BDD symptom severity, it has not been validated as a screening measure for BDD. Future studies should use tools specifically designed and validated for screening.

## Practical Implications

Our study assessed the reliability and validity of the FFITBS and MFBS within an Iranian population, providing clinicians and researchers with culturally appropriate tools to measure muscularity-related body dissatisfaction. University health services can utilize these scales to identify students who may experience body dissatisfaction and track the effectiveness of interventions over time. The FFITBS and MFBS demonstrated strong test–retest reliability and robust validity, making them ideal tools for longitudinal studies of body image in young adults and for future cross-cultural research. These image-based tools enable public health officials to screen the community for body dissatisfaction and to evaluate the effectiveness of body image prevention and intervention programs. Due to their minimal administrative and training requirements, the FFITBS and MFBS are valuable tools for school counselors and primary care providers, who can facilitate early detection and prevention in educational and clinical settings.

## Conclusion

This study validated the Persian version of the Fit Body Scale (FFITBS and MFBS) in an Iranian sample, demonstrating strong psychometric properties. The results confirmed good internal consistency, test-retest reliability, and acceptable concurrent, convergent, and divergent validity. The findings highlight the scale’s effectiveness in assessing muscularity-related body dissatisfaction in both men and women. Given its ease of administration and applicability across different populations, this tool can be valuable for research and clinical settings.

## Data Availability

The data supporting this study’s findings are available from the authors. However, their availability is restricted. These data were used under license for the current research and are not publicly available. However, the data are available from the corresponding authors upon reasonable request.
